# O Fim da "Sobrecarga do Átrio Esquerdo"

**DOI:** 10.36660/abc.20250814

**Published:** 2026-04-23

**Authors:** José Nunes de Alencar, Daniel Teixeira de Campos e Barreiro, Adrian Baranchuk

**Affiliations:** 1 Instituto Dante Pazzanese de Cardiologia São Paulo SP Brasil Instituto Dante Pazzanese de Cardiologia, São Paulo, SP – Brasil; 2 Faculdade de Medicina de Marília Marília SP Brasil Faculdade de Medicina de Marília, Marília, SP – Brasil; 3 Universidad Abierta Interamericana Bueno Aires Argentina Universidad Abierta Interamericana (UAI), Bueno Aires – Argentina

**Keywords:** Átrios do Coração, Eletrocardiografia, Diagnóstico

**Ao Editor,**

Lemos com grande interesse o artigo de Oliveira et al.^1^ Os autores devem ser elogiados por conduzirem um estudo desta magnitude e rigor. Enquanto a literatura anterior se baseava em amostras menores ou em valores de corte ecocardiográficos não padronizados/obsoletos para o alargamento do átrio esquerdo,^2–5^ a coorte ELSA-Brasil fornece o poder estatístico necessário para resolver um debate de longa data na cardiologia clínica. Acreditamos que esta pesquisa está destinada a se tornar um ponto de referência em livros-texto padrão de eletrocardiografia.

Gostaríamos também de abordar uma questão crítica de precisão terminológica e conceitual prevalente na literatura brasileira: o uso persistente do termo "Sobrecarga Atrial Esquerda" para descrever achados eletrocardiográficos. Acreditamos ser imprescindível que a comunidade cardiológica esteja ciente da diferença entre "alargamento" (uma alteração estrutural) e "sobrecarga" (um estado hemodinâmico). Como observado por Alpert et al., os critérios eletrocardiográficos frequentemente confundem erroneamente essas entidades fisiopatológicas distintas.^6^ Os autores do presente estudo utilizaram corretamente o índice de volume do átrio esquerdo (IVAE) por ecocardiografia como padrão ouro, que mede o alargamento (dilatação), e não a sobrecarga (carga de pressão ou volume). "Sobrecarga" implica um mecanismo hemodinâmico que o eletrocardiograma (ECG) não consegue medir diretamente, e o ecocardiograma o avalia apenas por meio de indicadores indiretos. Consequentemente, o termo "Sobrecarga" é cientificamente impreciso neste contexto e deve ser retirado do léxico em favor de termos que descrevam com precisão o fenômeno observado.

Se uma onda P ≥ 120 ms não prevê de forma confiável o alargamento do átrio esquerdo (AAE), muito menos a "sobrecarga", o que ela representa? A resposta está no conceito de bloqueio interatrial (BIA). Embora Josephson et al. tenham identificado corretamente que os padrões de "AAE" no ECG frequentemente representam atrasos de condução independentes do tamanho atrial.^7^ Foi o trabalho pioneiro de Bayés de Luna que transformou essa observação em uma entidade clínica distinta. A partir de 1979, ele descreveu e classificou sistematicamente esses distúrbios de condução, estabelecendo que o BIA poderia ocorrer transitoriamente e, crucialmente, na ausência de alargamento estrutural.^8^

Bayés de Luna foi além da mera observação da dissociação eletromecânica para estabelecer o significado clínico do achado. Ele classificou o BIA em formas parciais e avançadas — a primeira definida por uma onda P ≥ 120 ms com morfologia sinusal normal, enquanto este último também inclui morfologia bifásica (±) nas derivações inferiores — e identificou seu substrato patológico: cardiomiopatia atrial (CA) fibrótica.^9^ Essa pesquisa, que durou décadas, culminou no reconhecimento da "Síndrome de Bayés", identificando o BIA Avançado como um importante fator preditivo de arritmias supraventriculares.^10^

**Figura 1:**
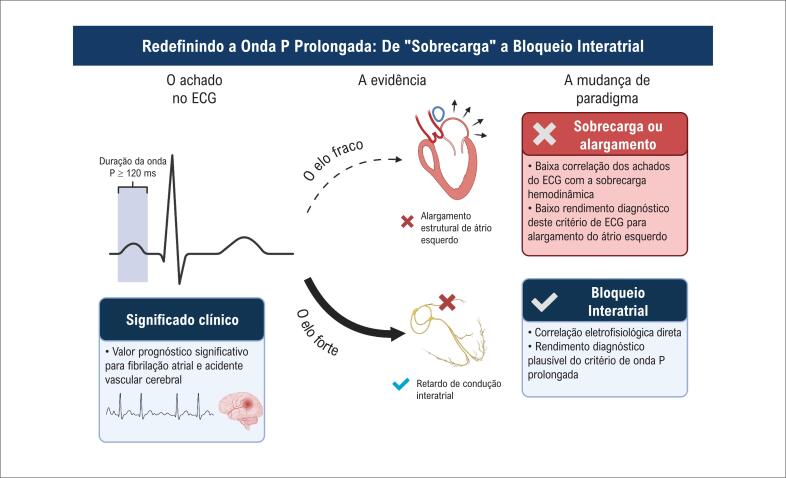
Embora uma onda P ≥ 120 ms seja tradicionalmente denominada "Sobrecarga Atrial Esquerda ou "Alargamento", evidências da coorte ELSA-Brasil (Oliveira et al.) demonstram uma fraca correlação com o alargamento estrutural real avaliado por ecocardiografia. Por outro lado, o achado elétrico representa um atraso de condução na região de Bachmann. Portanto, a nomenclatura deve mudar de suposições anatômicas ou hemodinâmicas ("Alargamento/Sobrecarga") para a definição eletrofisiológica precisa: Bloqueio Interatrial.

Portanto, uma duração da onda P ≥ 120 ms não deve ser considerada um "falso positivo" para alargamento, mas sim um "verdadeiro positivo" para BIA, especificamente um atraso de condução na região de Bachmann (Figura 1). Um limiar de 120 ms é o critério diagnóstico ideal para essa condição.^11^

Essa distinção tem um profundo peso prognóstico. O BIA é uma entidade independente associada à fibrose atrial e à remodelação eletromecânica.^12^ Além disso, estudos populacionais como o estudo ARIC^13^ demonstraram que esses defeitos de condução são preditores independentes de fibrilação atrial (FA). De forma semelhante à FA, esse marcador elétrico está fortemente associado a acidente vascular cerebral.^14^

Devemos abandonar o termo "Sobrecarga" porque ele implica uma sobrecarga hemodinâmica que não é medida diretamente pelo ECG nem é sinônimo das dimensões estruturais avaliadas pelo padrão ouro (IVAE). Além disso, dada a baixa acurácia demonstrada neste estudo, também devemos abandonar o termo "alargamento" para uma onda P prolongada. Em vez disso, a pesquisa de Oliveira representa uma excelente oportunidade para endossar uma distinção clara: classificar uma onda P ≥ 120 ms pelo que ela realmente é: BIA.^15^ Essa mudança terminológica garantirá a precisão científica e aprimorará a estratificação de risco para as arritmias e eventos embólicos associados à CA.
