# Supplementary material for: O Fim da "Sobrecarga do Átrio Esquerdo"
Source: Arq Bras Cardiol. 2026 Apr 23;123(4):e20250814. [Article in Portuguese] doi: 10.36660/abc.20250814 (PMC13155794; doi:10.36660/abc.20250814)
Supplement: Translation

# Translation: The End of "Left Atrial Overload"

**DOI:** 10.36660/abc.20250814i

**Authors:** José Nunes de Alencar, Daniel Teixeira de Campos e Barreiro, Adrian Baranchuk

**Affiliations:** 1 São Paulo SP Brazil Instituto Dante Pazzanese de Cardiologia, São Paulo, SP – Brazil; 2 Marília SP Brazil Faculdade de Medicina de Marília, Marília, SP – Brazil; 3 Bueno Aires Argentina Universidad Abierta Interamericana (UAI), Bueno Aires – Argentina

**Keywords:** Heart Atria, Electrocardiography, Diagnosis

**To the Editor,**

We read with great interest the article by Oliveira et al.^1^ The authors are to be commended for conducting a study of this magnitude and rigor. While previous literature relied on smaller sample sizes or non-standard/obsolete echocardiographic cutoffs for left atrial enlargement (LAE),^2–5^ the ELSA-Brasil cohort provides the statistical power necessary to settle a longstanding debate in clinical cardiology. We believe this research is destined to become a reference point in standard electrocardiography textbooks.

We would also like to expand upon a critical terminological and conceptual precision issue prevalent in Brazilian literature: the persistent use of the term "*Sobrecarga Atrial Esquerda*" (Left Atrial Overload) to describe electrocardiographic findings. We believe that it is imperative for the cardiology community to be aware of the difference between "enlargement" (a structural change) and "overload" (a hemodynamic state). As noted by Alpert et al., electrocardiographic criteria often mistakenly conflate these distinct pathophysiological entities.^6^ The authors of the current study correctly utilized left atrial volume index (LAVI) via echocardiography as the gold standard, which measures enlargement (dilatation), not overload (pressure or volume load). "Overload" implies a hemodynamic mechanism that the electrocardiogram (ECG) cannot directly measure, and the echocardiogram assesses it only through surrogates. Consequently, the term "Overload" is scientifically imprecise in this context and should be retired from the lexicon in favor of terms that accurately describe the observed phenomenon.

If a P-wave ≥ 120 ms does not reliably predict LAE, let alone "overload", what does it represent? The answer lies in the concept of interatrial block (IAB). While Josephson et al. correctly identified that "LAE" patterns on ECG often represented conduction delays independent of atrial size.^7^ It was the pioneering work of Bayés de Luna that transformed this observation into a distinct clinical entity. Beginning in 1979, he systematically described and classified these conduction disorders, establishing that IAB could occur transiently and, crucially, in the absence of structural enlargement.^8^

Bayés de Luna moved beyond the mere observation of electromechanical dissociation to establish the clinical significance of the finding. He classified IAB into partial and advanced forms—the former defined by a P-wave ≥ 120 ms with normal sinus morphology, while the latter also includes biphasic (±) morphology in inferior leads—and identified its pathological substrate: fibrotic atrial cardiomyopathy.^9^ This decades-long research culminated in the recognition of "Bayés Syndrome", identifying Advanced IAB as a key predictor of supraventricular arrhythmias.^10^

Therefore, a P-wave duration ≥ 120 ms should not be viewed as a "false positive" for enlargement, but rather as a "true positive" for IAB, specifically a conduction delay at the Bachmann region (Figure 1). A 120 ms threshold is the optimal diagnostic criterion for this condition.^11^

**Figure 1:** While a P-wave ≥ 120 ms is traditionally termed "Left Atrial Overload" (Sobrecarga) or "Enlargement," evidence from the ELSA-Brasil cohort (Oliveira et al.) demonstrates a poor correlation with actual structural enlargement assessed by echocardiography. Conversely, the electrical finding represents a conduction delay at the Bachmann region. Therefore, the nomenclature should shift from anatomical or hemodynamic assumptions ("Enlargement/Overload") to the precise electrophysiological definition: Interatrial Block.

This distinction carries profound prognostic weight. IAB is an independent entity associated with atrial fibrosis and electromechanical remodeling.^12^ Furthermore, population-based studies like the ARIC study^13^ have shown that these conduction defects are independent predictors of atrial fibrillation (AF). Similar to AF, this electrical marker is strongly associated with stroke.^14^

We should abandon the term "*Sobrecarga*" (Overload) because it implies a hemodynamic burden that is neither directly measured by the ECG nor synonymous with the structural dimensions assessed by the gold standard (LAVI). Furthermore, given the poor accuracy demonstrated in this study, we should also abandon the term "Enlargement" for a prolonged P-wave. Instead, Oliveira's research is a prime opportunity to endorse a clear distinction - label a P-wave ≥ 120 ms as what it truly is: Interatrial Block.^15^ This terminological shift will ensure scientific accuracy and enhance risk stratification for the arrhythmias and embolic events associated with atrial cardiomyopathy.
