# Supplementary material for: O Fim da "Sobrecarga do Átrio Esquerdo"
Source: Arq Bras Cardiol. 2026 Apr 23;123(4):e20250814. [Article in Portuguese] doi: 10.36660/abc.20250814 (PMC13155794; doi:10.36660/abc.20250814)
Supplement: Related texts

# Reply: Carta-resposta

**Authors:** Ana Cecilia Oliveira, Vinicius Tostes Carvalho, Marcelo M. Pinto-Filho, Luisa Brant, Sandhi Maria Barreto, Murilo Foppa, Antônio Luiz Pinho Ribeiro

**Affiliations:** 1 Universidade Federal de Minas Gerais Belo Horizonte MG Brasil Universidade Federal de Minas Gerais, Belo Horizonte, MG – Brasil; 2 Hospital de Clinicas de Porto Alegre Porto Alegre RS Brasil Hospital de Clinicas de Porto Alegre, Porto Alegre, RS – Brasil

Agradecemos imensamente os elogios ao nosso estudo na Carta ao Editor intitulada "O Fim da Sobrecarga do Átrio Esquerdo". Esperamos que nosso trabalho contribua para o conhecimento existente sobre anormalidades atriais.

Compartilhamos da posição expressa nesta Carta a respeito da recomendação contra o uso do termo "Sobrecarga", visto que implica sobrecarga de pressão ou volume, e, portanto, adotamos exclusivamente o termo "Alargamento". Em nosso artigo, conforme descrito na seção de Introdução, o alargamento do Átrio Esquerdo foi entendido como a terminologia atual para um padrão eletrocardiográfico que, na verdade, se enquadra no conceito mais amplo de CA. A CA corresponde a qualquer alteração estrutural, arquitetural, contrátil ou eletrofisiológica que afete os átrios com potencial para produzir manifestações clinicamente relevantes.^1^

Esta carta também trouxe uma discussão aprofundada sobre as origens e os critérios diagnósticos do BIA.^2^ Na seção de Discussão, indicamos o BIA como causa de onda P prolongada e descrevemos brevemente seus padrões eletrocardiográficos. Também destacamos o valor prognóstico da onda P prolongada, visto que ela está relacionada a desfechos clínicos adversos.^3–5^ A análise apresentada nesta carta expande significativamente o tema e certamente contribui para a discussão.

Além disso, o texto nos lembra que a eletrocardiografia não é uma ciência estática, mas dinâmica – uma ciência que deve ser continuamente debatida e atualizada. Atualmente, nos encontramos em meio a diversas mudanças paradigmáticas na eletrocardiografia. A crescente disponibilidade de exames de imagem e estudos eletrofisiológicos nos permitiu investigar mais a fundo as relações entre os achados eletrocardiográficos e seus presumidos mecanismos fisiopatológicos subjacentes. Esses avanços também evidenciaram que os padrões do ECG não refletem necessariamente anormalidades estruturais, o que, por exemplo, fundamenta o termo CA.^1^

Além disso, a introdução da inteligência artificial na interpretação do ECG levou à identificação de padrões anteriormente desconhecidos com significativo valor prognóstico, embora não necessariamente fundamentados no conhecimento eletrocardiográfico tradicional.^6^ Os avanços nessas áreas certamente desafiarão a terminologia atual e nos obrigarão, como médicos e cientistas, a rever conceitos bem estabelecidos.

Por fim, agradecemos a oportunidade de participar desta troca construtiva, que nos permitiu refletir mais sobre nossas descobertas e ampliar a discussão. Acreditamos que este diálogo não só aprofunda a compreensão do tema, como também ajuda a identificar direções promissoras para pesquisas futuras.

## References

[1] Chen LY, Ribeiro ALP, Platonov PG, Cygankiewicz I, Soliman EZ, Gorenek B, et al. P Wave Parameters and Indices: A Critical Appraisal of Clinical Utility, Challenges, and Future Research-A Consensus Document Endorsed by the International Society of Electrocardiology and the International Society for Holter and Noninvasive Electrocardiology. Circ Arrhythm Electrophysiol. 2022;15(4):e010435. doi: 10.1161/CIRCEP.121.010435.10.1161/CIRCEP.121.010435PMC907012735333097

[2] Luna AB, Platonov P, Cosio FG, Cygankiewicz I, Pastore C, Baranowski R, et al. Interatrial Blocks. A Separate Entity from Left Atrial Enlargement: a Consensus Report. J Electrocardiol. 2012;45(5):445-51. doi: 10.1016/j.jelectrocard.2012.06.029.10.1016/j.jelectrocard.2012.06.02922920783

[3] Magnani JW, Gorodeski EZ, Johnson VM, Sullivan LM, Hamburg NM, Benjamin EJ, et al. P Wave Duration is Associated with Cardiovascular and All-Cause Mortality Outcomes: the National Health and Nutrition Examination Survey. Heart Rhythm. 2011;8(1):93-100. doi: 10.1016/j.hrthm.2010.09.020.10.1016/j.hrthm.2010.09.020PMC304640120868770

[4] Yokota A, Kabutoya T, Hoshide S, Kario K. Automatically Assessed P-Wave Predicts Cardiac Events Independently of Left Atrial Enlargement in Patients with Cardiovascular Risks: The Japan Morning Surge-Home Blood Pressure Study. J Clin Hypertens. 2021;23(2):301-8. doi: 10.1111/jch.14136.10.1111/jch.14136PMC802981633340234

[5] Magnani JW, Johnson VM, Sullivan LM, Gorodeski EZ, Schnabel RB, Lubitz SA, et al. P Wave Duration and Risk of Longitudinal Atrial Fibrillation in Persons ≥ 60 Years old (from the Framingham Heart Study). Am J Cardiol. 2011;107(6):917-21.e1. doi: 10.1016/j.amjcard.2010.10.075.10.1016/j.amjcard.2010.10.075PMC304984921255761

[6] Lima EM, Ribeiro AH, Paixão GMM, Ribeiro MH, Pinto-Filho MM, Gomes PR, et al. Deep Neural Network-Estimated Electrocardiographic Age as a Mortality Predictor. Nat Commun. 2021;12(1):5117. doi: 10.1038/s41467-021-25351-7.10.1038/s41467-021-25351-7PMC838736134433816

# Reply

**Authors:** Ana Cecilia Oliveira, Vinicius Tostes Carvalho, Marcelo M. Pinto-Filho, Luisa Brant, Sandhi Maria Barreto, Murilo Foppa, Antônio Luiz Pinho Ribeiro

**Affiliations:** 1 Belo Horizonte MG Brazil Universidade Federal de Minas Gerais, Belo Horizonte, MG – Brazil; 2 Porto Alegre RS Brazil Hospital de Clinicas de Porto Alegre, Porto Alegre, RS – Brazil

We are extremely thankful for the generous compliments on our study in the Letter to the Editor entitled "The End of Left Atrial Overload". We hope that our work contributes to the existing body of knowledge regarding atrial abnormalities.

We share the position stated in this Letter concerning the recommendation against using the term "Overload," as it implies pressure or volume load, and have therefore adopted the term "Enlargement " exclusively. In our paper, as outlined in the Introduction section, left atrial enlargement was understood as the current terminology for an electrocardiographic pattern that, in fact, falls within the broader concept of atrial cardiomyopathy (AC). AC corresponds to any structural, architectural, contractile, or electrophysiological change affecting the atria with the potential to produce clinically relevant manifestations.^1^

This Letter also brought a thorough discussion on the origins and diagnostic criteria of IAB.^2^ In our Discussion section, we indicate IAB as a cause of prolonged P-wave and briefly describe its electrocardiographic patterns. We also pointed out the prognostic value of a prolonged P-wave, as it is related to adverse health outcomes.^3–5^ The review in this Letter significantly expands on the topic and certainly builds on the discussion.

Furthermore, the text reminds us that electrocardiography is not a static but a dynamic science - one that must be continuously debated and updated. At present, we find ourselves in the midst of several paradigm shifts in electrocardiography. The increasing availability of imaging and electrophysiologic studies has enabled us to further investigate the relationships between electrocardiographic findings and their presumed underlying pathophysiological mechanisms. These advances have also evidenced that ECG patterns do not necessarily reflect structural abnormalities, which, for instance, underpins the term atrial cardiomyopathy.^1^

In addition, the introduction of artificial intelligence in ECG interpretation has led to the identification of previously unknown patterns with significant prognostic value, although not necessarily rooted in traditional electrocardiographic knowledge.^6^ The advances in these fields will certainly challenge current terminology and compel us, as physicians and scientists, to review well-established concepts.

Finally, we are grateful for the opportunity to engage in this constructive exchange, which has allowed us to further reflect on our findings and broaden the discussion. We believe that this dialogue not only deepens the understanding of the topic but also helps identify promising directions for future research.
